# Global readiness for carbon neutrality: From targets to action^☆^

**DOI:** 10.1016/j.ese.2025.100546

**Published:** 2025-03-07

**Authors:** Shihui Zhang, Wenjia Cai, Xinzhu Zheng, Xuedu Lv, Kangxin An, Yuan Cao, Hou Sang Cheng, Jingyi Dai, Xinyang Dong, Shuting Fan, Yiying Gao, Zaizuo Gong, Yusheng Guan, Congkai Hong, Jie Li, Mingyu Li, Yukai Li, Songrun Liang, Weiyi Liao, Zhongqi Ma, Yue Ren, Jianxiang Shen, Xin Shi, Yang Su, Jinjie Sun, Chengqi Xia, Junyuan Xu, Wenxin Ye, Ling Zhang, Shangchen Zhang, Tianyi Zhang, Zihan Zhen, Hanying Zhong, Can Wang, Kebin He

**Affiliations:** a School of Environment, Tsinghua University, Beijing, 100084, China; b Institute of Carbon Neutrality, Tsinghua University, Beijing, 100084, China; c School of Ecology & Environment, Renmin University of China, Beijing, 100084, China; d Department of Earth System Science, Ministry of Education Key Laboratory for Earth System Modeling, Institute for Global Change Studies, Tsinghua University, Beijing, 100084, China; e School of Economics and Management, China University of Petroleum-Beijing, Beijing, 102249, China; f Tencent SSV Carbon Neutrality Lab, Shenzhen, 518054, China; g Schwarzman College, Tsinghua University, Beijing, 100084, China; h College of the Environment and Ecology, Xiamen University, Xiamen, Fujian, 361102, China; i Weiyang College, Tsinghua University, Beijing, 100084, China; j Institute of Energy, Environment and Economy, Tsinghua University, Beijing, 100084, China

**Keywords:** Carbon neutrality, Net-zero, Climate action, Global stocktake, Implementation gap

## Abstract

The global push for carbon neutrality highlights the need for rigorous assessments of whether national efforts align with stated targets. However, existing evaluations often prioritize commitments over tangible progress, lacking comprehensive and transparent metrics. To bridge this gap, we develop a multidimensional indicator system that evaluates targets, policies, actions, and effectiveness across key areas, including policy implementation, technology deployment, financial investment, and international cooperation. While 151 countries have pledged carbon neutrality—19 of which are developing nations that made commitments in 2024—implementation remains uneven. Only 72 countries have established complete policy frameworks, and advanced low-carbon technologies are concentrated in a handful of nations. Current trends indicate that global renewable energy capacity will reach just 2.7 times its 2022 level by 2030, falling short of the tripling target. Moreover, the global median action score in 2024 stands at only 25—far below the target of 65—highlighting the urgency for stronger efforts. Our findings reveal a significant gap between ambition and action, with renewable energy deployment lagging behind expectations. To accelerate progress, enhanced global cooperation, increased investment, and fewer barriers to technology diffusion are crucial. This study underscores the need for more implementation-focused tracking to ensure carbon neutrality commitments translate into measurable outcomes.

## Highlights

• Global carbon neutrality progress and implementation gaps are assessed using 170 indicators for 197 countries.
• The number of countries aiming for carbon neutrality increase from 133 in 2023 to 151 in 2024.
• Only 72 countries have a complete carbon neutrality policy frameworks.
• Current trends indicate renewable energy capacity may reach 2.7 times the 2022 level by 2030, missing the tripling goal.
• The global median action score stands at 25, far below the target of 65, highlighting the urgent need for more actions.

## Graphical abstract

**Figure:**
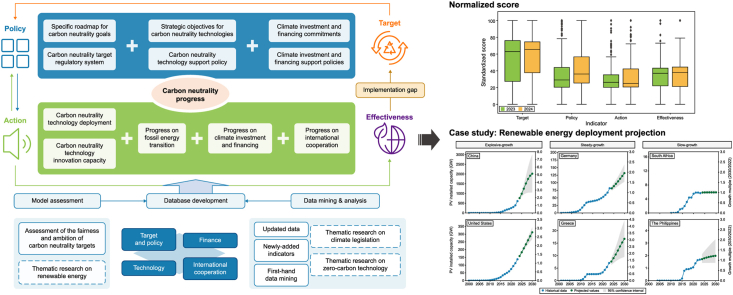

## 1 Introduction

With record-breaking global average temperatures and the increasing intensity, frequency, and scale of extreme weather events, mitigating climate change and transitioning to a net-zero carbon emissions (carbon-neutral) society is now a shared global goal. The Paris Agreement calls on nations to take concrete action to limit the global temperature increase to well below 2 °C by the end of the century [1]. As of May 2024, 148 countries had made explicit net-zero or carbon neutrality commitments, and all 198 parties to the United Nations Framework Convention on Climate Change (hereinafter the ‘Convention’) had enacted laws on climate change [2]. The 28th Conference of the Parties (COP28) to the Convention conducted its first global stocktaking, calling for the tripling of global installed renewable energy capacity by 2030 [3].

The focus of the global climate agenda has now shifted from goal setting to implementing actions. Governments are increasingly adopting more ambitious emission reduction targets and stricter policies. However, translating these commitments into tangible progress requires effective implementation of technologies, financial instruments, and international cooperation—key issues in ongoing climate negotiations. Disparities in natural resources, socio-economic development, and technological capacities continue to hinder the effective execution of climate policies. Despite ambitious targets, the slow pace and limited scale of renewable energy development in many countries make it difficult to meet goals, such as achieving a specific percentage of renewable capacity in the power grid by a set year [4]. Furthermore, progress relating to global climate finance and international cooperation has been slow [5], impeding the expansion of low-carbon technologies to developing countries [6,7]. To address these challenges, comprehensive tracking of progress from carbon neutrality goals to actions and identification of the gaps between current mitigation efforts and ambitious carbon neutrality goals are crucial.

Responding to the above needs, several renowned research institutions have tracked and reported on the status of carbon neutrality commitments, providing valuable insights into the ambitions underlying targets in key countries and their legal effectiveness [[8], [9], [10], [11]]. However, these reports contain significant gaps. For instance, the criteria used to evaluate countries' commitments often overlook critical factors such as per capita emissions, historical responsibility, and each country's emission reduction capacities. Moreover, while they focus heavily on targets, they are often less action-oriented, providing qualitative assessments of policies that lack a systematic, objective framework for tracking the implementation of the actions required to meet those targets. In addition, the reports primarily focus on major emitters, notably the G20 and emerging economies, with limited attention given to smaller or less developed nations. These gaps underscore the need for a more comprehensive, systematic approach for monitoring global progress towards carbon neutrality that provides broader geographic coverage and deeper analysis of critical factors—technology, finance, and international collaboration driving the transition.

**Fig. 1:**
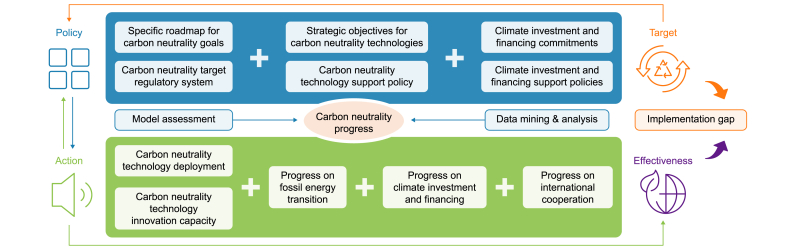
Analytical framework of the study.

Our report presents a comprehensive analysis of global progress towards carbon neutrality targets, policies, and actions, covering multiple dimensions, such as targets, technologies, finances, and international cooperation (Supplementary Material Table S1). We also assessed the effectiveness of carbon neutrality transitions by countries and summarized the characteristics of the transition into four different types. While many studies have tracked these dimensions separately, this study contributes to the literature by integrating them into a unified indicator system, which enables evaluations of the progress of each country relating to its carbon neutrality performance across four key stages: target–policy–action–effectiveness. ‘Target’ encompasses the scope, ambition, and specificity of climate commitments, such as sectoral coverage or subnational goals. ‘Policy’ covers supportive regulations, financial tools, and governance systems, such as subsidies, mandates, or roadmaps. ‘Action’ refers to measurable progress indicators. Including renewable energy deployment, fossil fuel phase-down, and mitigation budgets. ‘Effectiveness’ refers to the pace and scale of emission reductions achieved, adjusted for differentiated implementation challenges by countries (Fig. 1). This methodology advances understanding of the implementation gap between pledged goals and actual progress [12].

The analysis for the 2024 annual progress report on global carbon neutrality is underpinned by extensive and wide-ranging data sourced from international organizations, government websites, scientific research publications, and the reports of multilateral agencies. A comprehensive database was created, featuring 17 primary indicators, 55 secondary indicators, and 170 tertiary indicators organized according to four critical dimensions: targets, technology, finance, and international cooperation. This open-access database, which is available on the Global Carbon Neutrality Tracker website [13], provides a transparent, systematic tool for tracking the progress of efforts towards achieving carbon neutrality in over 190 countries.

The report also presents advanced model-based analyses for evaluating gaps between global carbon neutrality targets and actual implementation. Using historical and projected data on emissions, gross domestic product (GDP), and population, we modelled future emission pathways under 1542 fair distribution scenarios, including approaches based on responsibility, capacity, per capita emissions, and statism [14]. These modelling techniques were used to calculate an ambition index (0–1). This metric quantifies the probability that a country's emission reduction targets are equitably aligned with the global emissions budget required to limit global warming to 1.5 °C, with a score of 1 indicating full compliance. Projections of renewable energy capacity through to 2030 were made by combining the Richards model [15] with Markov chain Monte Carlo (MCMC) Bayesian inference [16]. The Richards model is a flexible generalized logistic model that outperforms traditional S-shaped curves by incorporating an additional parameter better suited for bounded growth patterns like renewable adoption. MCMC Bayesian inference is a probabilistic framework that rigorously explores uncertainties in model parameters to generate forecasts with robust confidence intervals. We also assessed the impact of tariff changes on the international renewable energy trade, especially solar photovoltaic (PV) and wind turbine technologies, to elucidate how trade policies influence the global diffusion of low-carbon technologies. Lastly, we evaluated the economic and social benefits accrued from foreign renewable energy investments in developing countries using input-output models to quantify their impacts on local economies, job creation, and energy access. Detailed methodological explanations for the approaches used are provided in subsequent sections.

The 2024 report aimed to answer the following key questions: (1) What were the latest developments and trends in global carbon-neutral targets, policies, and actions in 2023? (2) How effective is the global transition toward carbon neutrality, and what action gaps remain to be addressed to achieve this goal worldwide? (3) Are renewable energy and other critical zero-carbon technologies advancing sufficiently rapidly to support global carbon neutrality? What new opportunities and challenges exist relating to the deployment and diffusion of these technologies? (4) What legislative progress has been made by countries towards attaining carbon neutrality? Are commitments backed by robust legal systems and effective policy implementation? (5) How can the gap between carbon neutrality targets and actual actions be further narrowed to strengthen international cooperation, foster technological innovation, and enhance climate finance that ultimately support a global process to achieve carbon neutrality and a just transition?

This report tackles these questions by tracking the annual progress towards carbon neutrality in 2024 across several key areas. These areas are: targets and policies (section 2), technologies (section 3), finance and investments (section 4), international cooperation (section 5), and national progress scores (sections 6, 7). Section 8 presents further analysis and policy recommendations, and section 9 offers conclusions.

## 2 Progress towards global carbon neutrality targets and legal support systems

### 2.1 Progress in target setting

**Fig. 2:**
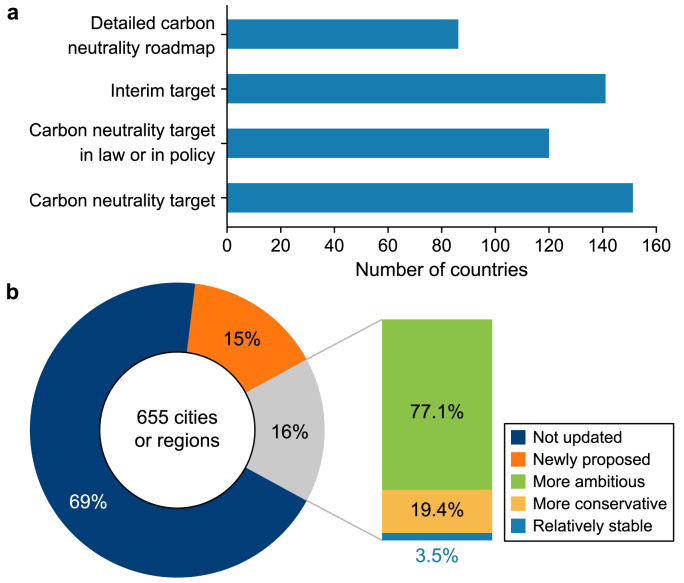
**a**, Global carbon neutrality targets and corresponding support systems. **b**, Progress towards city or regional carbon neutrality targets.

Global coverage of carbon neutrality targets is gradually expanding and transitioning into the implementation phase. Between June 2023 and May 2024, the number of countries with carbon neutrality targets rose from 133 to 151, with developing countries accounting for all the new targets. In addition to countries that have explicitly set carbon neutrality targets, two countries, the Netherlands and Norway, have set a goal of reducing emissions by 95 % relative to the 1990 level, with plans to offset the remaining emissions through carbon offset mechanisms. Following the principle that ‘actions speak louder than words’, we categorized the emission reduction targets of these two countries as carbon neutrality targets. As of May 2024, of the 151 countries that had proposed carbon neutrality targets, 120 had implemented laws to establish the legal status of carbon neutrality targets, or they had introduced relevant policies, with significant policy contributions made by developing countries. Additionally, 86 countries have proposed detailed roadmaps for achieving carbon neutrality (Fig. 2a). More than 90 % of countries that have not yet set a carbon neutrality target have already established carbon emission reduction targets. It is expected that more countries will propose carbon neutrality targets during the next round to update Nationally Determined Contributions (NDCs), indicating an irreversible global trend towards carbon neutrality target setting.

Carbon neutrality targets at the regional and city levels have become more ambitious. Globally, 655 cities or regions have set carbon neutrality targets. The targets’ coverage is continually expanding, with 99 new cities or regions having set carbon neutrality targets from June 2023 to May 2024 (Fig. 2b). Moreover, 103 cities or regions have updated their targets, with nearly half adopting more ambitious goals relating to the target type, year, or legal commitment. Of those that updated their targets, 77.1 % have set more ambitious carbon neutrality targets.

Some countries and regions also experienced policy setbacks. Of those that updated their carbon neutrality targets, 19.4 % have regressed on their goals, with 11 regressions (55 %) occurring in developed countries. Setbacks mostly occurred in South Asian and European countries. The British government has postponed the cut-off date for the production of new gasoline and diesel cars from 2030 to 2035. The Mexican government eliminated policies supporting renewable energy and is instead prioritizing fossil fuels. Germany has delayed the implementation of heating systems that operate entirely on energy sources that are greenhouse gas (GHG)-neutral. Some developed countries have even withdrawn from the international climate finance cooperation initiative.

**Fig. 3:**
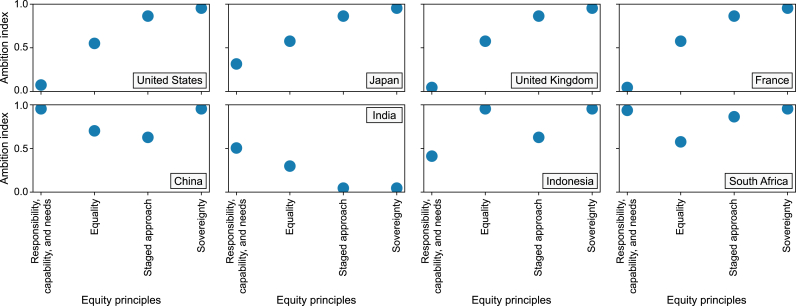
Ambition index values or major countries in meeting the 1.5 °C target under different fairness principles.

According to model assessments, almost all of the 151 countries that proposed carbon neutrality targets have made the efforts required to keep the global temperature rise below 2 °C. The strength of each country's carbon-neutral target was assessed by modelling whether it fell within the global emissions budget under the 2 °C/1.5 °C target. The ambition index of each country's target was quantitatively assessed to determine if it meets the requirements of the 2 °C/1.5 °C target under different equity principles (Fig. 3). The ambition index refers to the probability that a country's target is aligned with the allocated emissions budget under the 1.5 °C target according to certain equity principles. It ranges from 0 to 1, with a score of 1 indicating that each country's cumulative carbon emissions are consistent with its allocated budget under all different allocation principles for the 1.5 °C target. In line with the principle of historical responsibility and emissions reduction capacity, developing countries had a higher ambition index, reflecting greater willingness to reduce emissions despite their limited emissions reduction capacities. By contrast, developed countries had a lower ambition index according to the above principle and their emission reduction capacities, but their ambition index was higher according to the principles of statism and phased equity. Thus, their emission reduction targets did not match their historical responsibility and national capability, and they evidently prioritized their own developmental interests.

### 2.2 Progress of legal support systems

Climate legislation has become increasingly important for supporting carbon neutrality targets. Globally, 35 countries have enshrined carbon neutrality targets in their laws, and all parties to the Convention have enacted legislation that responds to climate change. Climate litigation is mainly concentrated in high-income countries. Developing countries have demonstrated remarkable progress in enacting renewable energy legislation, with most laws introduced in low- and middle-income countries, particularly in Southeast Asia.

**Fig. 4:**
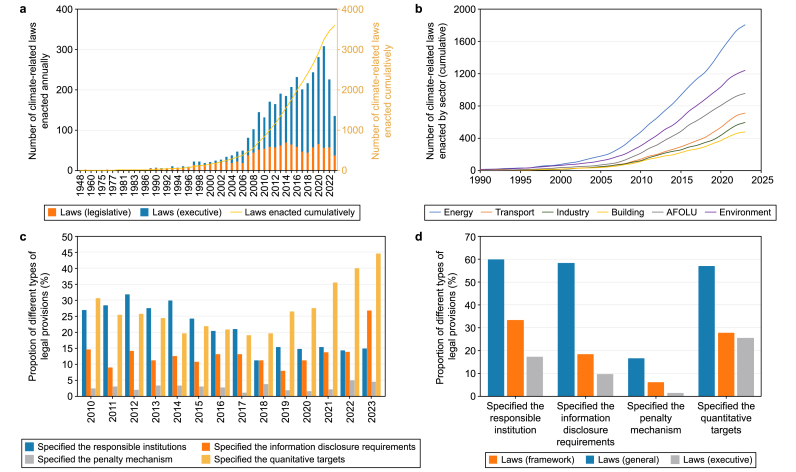
**a**, Global climate-related legislation: growth trends and type distribution. Number of climate-related laws enacted annually/cumulatively. **b**, Number of climate-related laws enacted by sector (cumulative). AFOLU: agriculture, forestry, and land use. **c**, Percentage of each type of article relative to all articles enacted annually from 2010 to 2023. **d**, Percentage of each type of article in framework laws, legislative laws, and executive regulations.

International climate negotiations and the commitments of major countries have significantly impacted climate legislation. As of June 2024, more than 3600 climate-related laws had been enacted globally, covering all 198 Convention Parties. Fig. 4a shows the gradual increase in the number of climate-related laws enacted each year since the dawn of the 21st century. Following the Paris Agreement, the number of climate-related laws enacted in 2016 exceeded 220, reaching the first peak, and related growth was mainly attributed to administrative regulations. During 2020–2021, driven by China's ‘dual-carbon’ commitment, the European Green Deal, and re-ratification of the Paris Agreement by the United States, global climate action accelerated, and the number of related laws and administrative regulations grew significantly.

The energy sector has consistently led the way, with the enactment of over 1800 energy-related laws. Fig. 4b shows that prior to 2015, global climate action was dominated by ‘conservation efforts’, with the environmental and agriculture, forestry, and land use (AFOLU) sectors ranked second after the energy sector for the number of enacted laws. Following the ratification of the Paris Agreement, the number of laws enacted annually in the transport, industry, and building sectors increased by 40–60 % compared with the averages for the years before 2016. In 2023, for the first time, laws related to the transport sector outnumbered those on the environment and AFOLU sectors. In developed, Western European countries such as the United Kingdom (UK), Germany, and Sweden, up to 80 % of climate laws are related to the energy, industry, transport, and building sectors, which is 5–20 % higher than the figures for most developing countries. Climate laws enacted in rainforest-rich countries, such as Brazil, Vietnam, and India, have significantly higher proportions of AFOLU and environment-related laws, together accounting for nearly 50 % of total enacted laws.

Since the adoption of the Paris Agreement, implementation has received greater emphasis within national climate legislation, with a significant increase in the number of administrative regulations enacted each year. The number of administrative regulations enacted in 2016 exceeded 150 (Fig. 4a), which represents a 25 % increase over the averages for the years before 2016. The proportion of climate laws and regulations with quantitative targets and information disclosure requirements has grown rapidly. From 2018 to the present, laws specifying quantitative targets increased from 20 % to 40–50 %, while those specifying information disclosure rose from <10 % to approximately 25 % (Fig. 4c).

A total of 65 climate change legal frameworks have been enacted in 57 countries, 70 % of which are developed countries, mainly located within Western Europe and the Americas. To date, only 19 developing countries have enacted climate change legal frameworks. China, the United States, and India are yet to enact their framework law. Between 50 % and 60 % of the climate change legal frameworks specify responsible institutions, information disclosure requirements, and quantitative targets (Fig. 4b).

## 3 Progress relating to the development of global carbon neutrality technology

### 3.1 Progress in renewable energy development and the feasibility of the ‘tripling goal’

Renewable energy is a core driver of global carbon neutrality. Therefore, it is crucial to assess whether its current pace of deployment is aligned with the COP28 target of tripling capacity. The global ambition for renewable energy development continues to rise, with nearly two-thirds of countries having proposed targets. By the end of 2023, 133 countries had proposed renewable energy power generation targets, reflecting an increase of 23 countries from 2022. Among them, the number of countries that have set a 100 % renewable energy power generation target increased from 30 in 2022 to 37, with Ecuador, Nepal, Tuvalu, and other developing countries being the main additions.

**Fig. 5:**
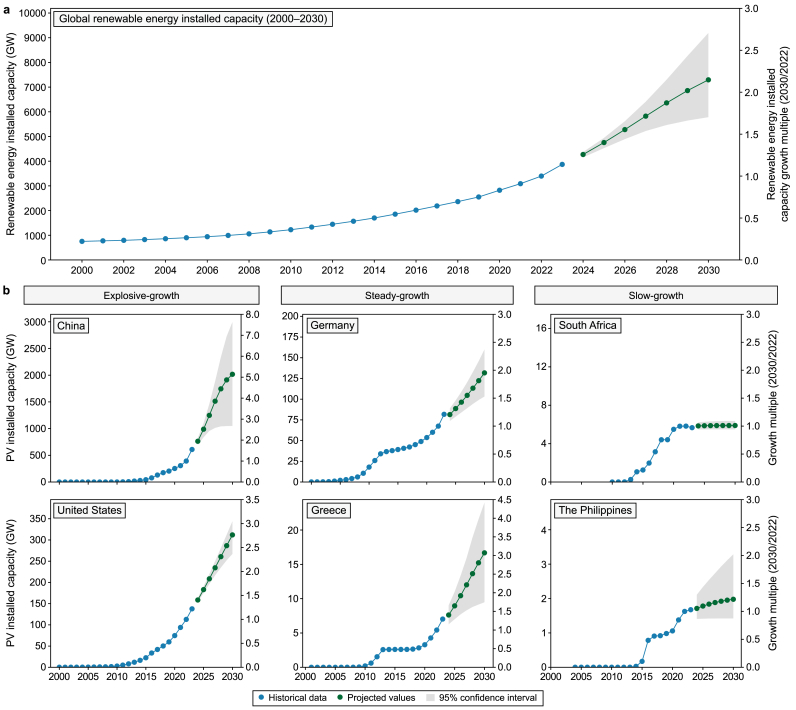
**a**, Historical trend (2000–2023) and future projection (2024–2030) of global renewable energy installed capacity growth. **b**, Historical trend and future projection of installed photovoltaic (PV) capacity growth in typical countries. The blue scatter points represent historical data, while the green ones indicate projected values. The shaded gray area denotes the 95 % confidence interval. The secondary *y*-axis shows the growth multiple corresponding to the installed capacity.

If the historical growth trend from 2000 to 2023 persists, installed renewable energy capacity globally will only be 1.7–2.7 times higher than the 2022 level by 2030, and the probability of achieving the tripling target will be extremely low. Meeting this target by 2030 would require a global installed renewable energy capacity of 11,000 GW. If the growth trend during the last two decades persists, the global capacity will only reach 5800–9200 GW by 2030, which is 1.7–2.7 times higher than the 2022 level (Fig. 5a). Among the six best-performing countries, only China is expected to reach an installed renewable energy capacity that is 3.5 times higher than the 2022 level by 2030, while the remaining five countries (the United States, Brazil, India, Germany, and Japan) are unlikely to exceed 2.5 times the 2022 level.

Given the growth rate of renewable energy in 2023, exemplified by wind and solar PV, which far exceeded historical levels, the tripling target is likely to be achieved if industrial support policies and technological advancements are strengthened. From June 2023 to May 2024, new global renewable energy installations evidenced 14 % year-on-year growth. By the end of 2023, the global installed capacity of renewable energy had reached 3870 GW, accounting for about 43 % of the total global installed power capacity. Solar PV and wind power saw significant increases, with new installed capacity of 346 GW and 116 GW, up by 32 % and 13 %, respectively, year-on-year. In light of the current industrial policies and accelerated technological advances, accompanied by the rising rate of renewable energy installation, the probability of meeting the tripling target is increasing.

Three types of renewable energy trends can be identified worldwide according to historical growth trends: explosive growth, steady growth, and slow growth (Fig. 5b). Solar PV and wind power have shown average annual growth rates of 30–60 % over the last three years in countries experiencing explosive growth, such as China, the United States, and Indonesia, and are expected to more than triple their current installed capacity by 2030. The main drivers of this growth are industrial policies like Feed-in Tariff (FIT) schemes and declining renewable energy costs attributed to technological advancements. Average annual growth rates of solar and wind power in steady-growth countries, such as Germany, Greece, and other developed European nations, and some member countries of the Organization for Economic Cooperation and Development (OECD) are 5–15 %. These countries rapidly adopted renewable energy around 2010, with installed capacities accounting for nearly 70 % of the total global capacity at one time. However, the growth rate declined between 2015 and 2020 because of less favourable industrial policies. In recent years, new policies and technological advances have prompted renewed growth in some countries, but without stronger measures, their installed capacity will only increase by 1.3–2.0 times the current level by 2030. Slow-growth countries are mainly concentrated in less developed regions of Africa and Latin America, whose renewable energy production is characterized by discontinuous and unstable growth and accounts for approximately 20 % of the total global production. For example, in 2023, the growth rate of solar PV in Africa was just 5 %, being significantly lower than the average growth rates of other continents (approximately 20–40 %).

Achieving the global target of tripling renewable energy capacity will require concerted international efforts and country-specific measures. Explosive-growth countries should continue to maintain high growth rates through technological innovation and policy optimization. Steady-growth countries need to strengthen their policy interventions by reintroducing industry incentives, such as subsidies and tax breaks, while promoting international cooperation and financing mechanisms to attract private capital. Slow-growth countries, especially those in Africa and Latin America, urgently require financial support, technology transfers, and policy assistance provided by the international community. Multilateral institutions and international climate funds should increase investments in these regions and promote global cooperation to ensure the realization of global renewable energy targets.

### 3.2 Deployment status of zero-carbon technology and implementation gaps

Several developing countries have proposed new, ambitious, zero-carbon technology deployment targets, with global light-duty electric vehicle sales evidencing >30 % growth and the numbers of green hydrogen and carbon capture, utilization and storage (CCUS) projects increasing by approximately 40 % and 110 %, respectively. In 2023, 23 new countries, which were mostly developing countries, proposed targets for electric vehicle shares, and 15 new countries proposed quantitative targets for green hydrogen deployment. Nearly 30 countries have proposed ambitious targets for 100 % zero-emission vehicle sales or bans on the sale of fuel-powered passenger cars, and >60 countries (an increase from 22 in 2022) have proposed national hydrogen strategies. In 2023, global sales and ownership of light-duty electric vehicles exceeded 13 million and 39.6 million units, respectively, reflecting increases of 30 % and 50 % relative to figures for 2022. Over 1800 green hydrogen projects (an increase of >500 projects from 2022) were planned globally. Nearly 200,000 tonnes year^−1^ of green hydrogen capacity were commissioned, quadrupling from the 2022 level. Over 90 % of China's green hydrogen projects commissioned in hard-to-abate sectors, such as transport and industry, including the world's largest (260 MW) green hydrogen project in Kuqa, Xinjiang, received final investment approval. By contrast, green hydrogen projects in Western European countries, such as Germany and Spain, were small-scale and characterized by diversification, with approximately 30 % of the projects focusing on emerging sectors such as power and construction, and almost 40 % of the projects having multiple downstream applications. The number of planned CCUS projects increased by 49.8 % from 162 in 2022 to 350 in 2023, demonstrating a total capture capacity of 361 million tonnes year^−1^. CCUS projects in the United States were more diverse and included hard-to-abate sectors such as iron and steel, cement, and chemicals. Denmark, Norway, and other Nordic countries were more inclined towards the use of bioenergy with carbon capture and storage (BECCS) technology, with 30–40 % of CCUS projects focusing on biomass and heat generation.

**Fig. 6:**
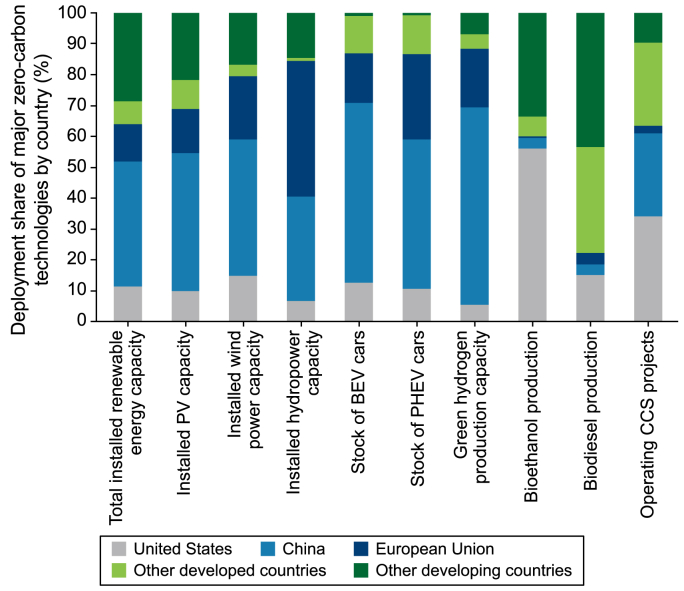
Deployment shares of major zero-carbon technologies by country. PV: photovoltaic; BEV: battery electric vehicle; PHEV: plug-in hybrid electric vehicle; CCS: carbon capture and storage.

The global deployment of zero-carbon technologies exhibited a clear regional concentration, with China, the United States, and the European Union (EU) collectively accounting for 70–90 % of global deployment in several zero-carbon technology areas. China, the United States and the EU respectively accounted for 40 %, 11 %, and 12 % of the total global installed renewable energy capacity (Fig. 6). Moreover, they collectively accounted for nearly 90 % of global electric vehicle ownership and green hydrogen capacity, with China contributing 50–65 % of this total. However, developed countries accounted for 60–70 % of the total global biofuel production and CCUS projects, with these technologies dominated by the United States. By contrast, developing countries other than China accounted for <30 % of various deployed technologies. Low-carbon technology diffusion is evidently crucial to achieve carbon neutrality worldwide, with cooperation being emphasized in the policy recommendations.

The promulgated global green hydrogen capacity target will only achieve half of the required scale of deployment under the global 2050 net-zero emissions (NZE) target. Even if all of the announced projects are completed, the global green hydrogen and CCUS capacity is expected to reach 40–50 % of the required scale to meet the NZE target by 2030. According to the International Energy Agency (IEA) projection [17,18], under the NZE target, the global green hydrogen capacity should reach 50–55 million tonnes year^−1^, and the CO_2_ capture and sequestration capacity should reach 1 billion tonnes year^−1^ by 2030. The statistics presented in this report reveal that the combined 2030 green hydrogen capacity target announced by countries is approximately 27 million tonnes year^−1^, which is only half of the required scale for meeting the NZE target. Moreover, only a few countries, notably the United States and Japan, have proposed specific CO_2_ capture targets. The commissioned green hydrogen capacity, CO_2_ capture, and sequestration capacity have respectively reached just 0.4 %, 4.9 % and 6.5 % of the required scale under the NZE target for 2030. If all of the announced projects are completed, the actual global green hydrogen and CO_2_ capture capacity are projected to reach 27 million tonnes year^−1^ and 430 million tonnes year^−1^, respectively by 2030, which would meet 40–50 % of the corresponding demand under the NZE target. However, this potential capacity remains highly uncertain, with final investment decisions reached for <15 % of projects. Countries worldwide will need to accelerate their deployment of relevant technologies to increase the rate of project implementation.

## 4 Progress in climate finance and investment supporting carbon neutrality

Sufficient finances are essential for achieving global carbon neutrality, but inadequate investment data hinders effective action. This section explores information disclosure, policies, and the gap between climate finance demand and supply.

### 4.1 Climate finance information disclosure

**Fig. 7:**
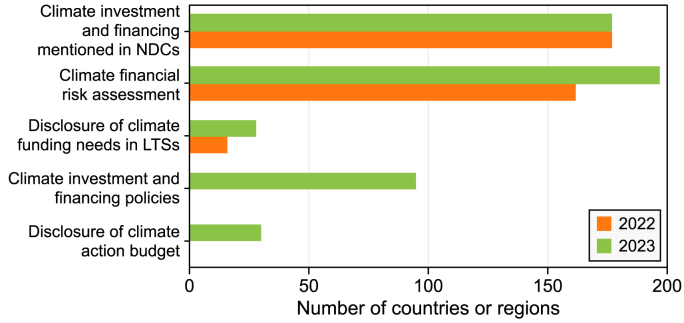
Numbers of countries or regions with different climate investment schemes and varying financing information disclosure (2022 vs. 2023). The information for the first three indicators was derived from the nationally determined contributions (NDCs) submitted by individual countries, and the information for the last two indicators was newly obtained from the public documents released by different countries in 2023, with 2022 data not yet available for comparison. LTSs: long-term low greenhouse gas emission development strategies.

In 2023, there was a moderate increase in the number of countries conducting climate finance needs and risk assessments. From 2022 to 2023, the number of countries reporting climate finance remained stable at 177 out of >190 countries that submitted or updated their NDCs (Fig. 7). The number of countries that disclosed information on their climate finance needs in their long-term low GHG emission development strategies (LTSs) rose from 16 to 28. The number of countries that conducted climate-related financial risk assessments also rose from 162 to 197, with 34 of the 35 new countries being developing countries. Despite this progress, most countries are yet to provide full disclosure of their climate action expenditure in their national budgets, and only 30 countries have provided accessible disclosure information. New Zealand and South Africa currently lead in the area of information disclosure.

### 4.2 Climate finance policy

The introduction of national climate finance policies remains limited, and carbon prices among countries or regions continue to be insufficient. Currently, only 95 countries have implemented climate finance policies. In 2023, several countries or regions expanded the coverage of their carbon pricing mechanisms, which are considered a mature climate finance policy instrument, with increases in both numbers of mechanisms and carbon price levels observed worldwide. From 2022 to 2023, the number of carbon pricing mechanisms increased from 72 to 73. Five countries or regions introduced new carbon pricing mechanisms, while four were disbanded. These mechanisms now cover 23.11 % of global GHG emissions. In 2023, the global emission-weighted carbon price was USD 14.2 per t CO_2_e, up by USD 0.7 per t CO_2_e from 2022, with the highest global carbon prices recorded for Uruguay and the EU. According to the World Bank's estimations, only 7 of the 73 global carbon pricing mechanisms (10 %) have prices that exceed the level for meeting the 2 °C target (USD 63–127 per t CO_2_e) [19].

### 4.3 Climate investment needs and gaps

There is a significant gap between the global need for climate investment and finance and actual investments, with developing countries facing particularly severe shortfalls. According to the Sixth Assessment Report of the Intergovernmental Panel of Climate Change (IPCC) [20], the annual global investment required to achieve mitigation and carbon neutrality is estimated at USD 2.3–4.5 trillion. To achieve the 2 °C target, global investments in climate mitigation must increase by a factor of three to five. This is particularly crucial for least developed countries, where the need is even more urgent, requiring an increase that is four-to seven-fold. Integrating climate action into national budgets is a crucial aspect of national climate governance. However, these budgets remain insufficient, and in some countries, they have not been fully realized. Among the countries that have disclosed their climate budgets, some developed countries, such as France and Germany, are leading the way, allocating 8–10 % of their total budgets. Despite their small overall budgets, Small Island Developing States, such as Dominica and Beliz, have performed well regarding the proportion allocated for climate funding. Despite the upward global trend in renewable energy investments, over 90 % of funding is concentrated in Europe, North America, East Asia, and the Pacific, while least developed countries receive less than 1 % of global renewable energy investments [21]. The costs of financing carbon-neutral technologies in least developed African countries remains significantly higher than those in developed countries, creating a severe mismatch between needs and capacities. Despite intense discussions on the new collective quantified climate finance goal (NCQG) at COP 29, a concrete and viable financial goal remains elusive.

## 5 Progress in international cooperation to promote carbon neutrality

### 5.1 International technology transfers

The number of international technology transfer projects has increased, but the proportion of direct technical support evidences a marked decline. According to the national biennial transparency reports submitted to UNFCCC, the total number of projects disclosed in the latest biennial report increased by 45 % compared with those in previous reports before 2023. However, the proportion of projects providing direct technical support declined from 50 % to 20 % in the previous report before 2023. The current disclosure of international technology transfer projects has been completely dominated by ‘soft support’ projects (capacity building, training, etc.). To enhance the quality of the projects, the level of disclosure and assessments of their effectiveness should be strengthened. Effective renewable energy technology transfer projects can help some African countries that are in the early stages of economic growth and energy transition speed up the start-up period for renewable energy deployment and synergize their low-carbon transition with economic growth and job security.

### 5.2 International climate finance

While the scale of developed countries' financial commitments to international climate finance has increased, it remains below the USD 100 billion contribution target. Developed countries’ commitments to climate finance have increased, and they were expected to provide USD 94.7 billion annually for international climate finance by 2025. However, existing commitments are insufficient for meeting the USD 100 billion funding target. In terms of the funding modality and content, only one-quarter of funds are direct donations, with loans, which are primarily non-concessional, accounting for the remainder. Direct climate net aid accounts for only one-quarter of the total amount of funding (approximately USD 20 billion per annum) [5]. On a country-by-country basis, Japan, France, Germany, the UK, and Italy have performed relatively better in terms of their funding commitments.

### 5.3 International investments and trade

The upward revision of tariffs on key renewable energy products in some countries could hinder the global diffusion of low-cost carbon-neutral technologies and increase the cost of global actions towards achieving carbon neutrality. Foreign direct investment (FDI) plays an important role in accelerating renewable energy deployment. According to the World Investment Report from United Nations Conference on Trade and Development (UNCTAD), international project finance accounts for 55 % of renewable energy investment across countries, with least developed countries accounting for >75 % of investments [22]. From 2010 to 2021, direct and indirect economic outputs from renewable energy FDIs amounted to 1.09–1.67 times the investment and created >0.2 million jobs. However, increases in import tariffs on key renewable energy components in some countries have not only raised the cost of carbon-neutral technology transfers, but have also slowed down the progress of technology cost declining. International trade is critical for the diffusion of technology for solar and wind power generation in developing countries, with the potential to save tens of billions of dollars annually in those countries and accelerate a just global transition.

## 6 Progress in carbon neutrality scores globally

**Fig. 8:**
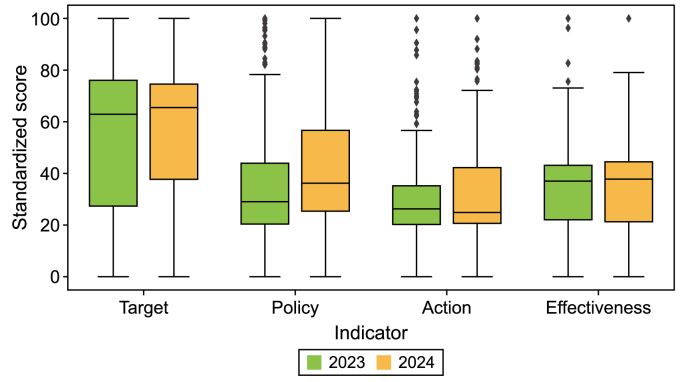
Distribution of carbon neutrality target-policy-action-effectiveness scores by country in 2023 and 2024. Some scores exhibit a significant deviation from the overall distribution and are identified as outliers, marked with diamond shapes.

Every country received a score from 0 to 100 across four dimensions: target setting, policy design, zero-carbon actions, and emission reduction effectiveness. These scores were derived from a normalized process, wherein weights were assigned to the relevant indicators in the database (Supplementary Material Table S2). A perfect score of 100 for target setting reflected the most ambitious and globally aligned climate goals. The scores for policy design and zero-carbon actions reflected the most advanced countries' progress relating to policy development and the implementation of zero-carbon initiatives. The score for emissions reduction effectiveness assessed the actual speed and impact of carbon reductions, considering the different challenges that countries face in reducing emissions and ensuring that the scores were contextually fair and reflective of each country's specific circumstances. The median scores obtained were 65 for targets, 36 for policies, 25 for actions, and 38 for effectiveness (Fig. 8). These scores reveal that countries are approaching optimal practices for achieving carbon neutrality targets, but policies, actions, and effectiveness still require substantial improvement.

### 6.1 Global carbon neutrality target scores

New targets in developing countries and wider coverage of targets in developed countries are driving progress globally, raising the global target score to 65 (compared with the median score of 61 in 2022). A total of 19 developing countries have set new carbon neutrality targets for significantly narrowing the global gap. Developed countries scored higher for the target type and coverage but fell short regarding the intensity and equity of targets, whereas developing countries showed the opposite trend.

### 6.2 Global carbon neutrality policy and action scores

Significant progress has been made in implementing carbon neutrality policies, but actions remain uneven, with particularly large gaps in climate financing and technological innovation. The number of countries with comprehensive carbon neutrality policy frameworks increased from 55 to 72 and included 38 developing countries. Kenya, for instance, has introduced a national sustainable finance strategy, carbon neutrality plans for multiple cities, and a relatively complete legal regulatory framework. However, in general, climate financing policies remain a major bottleneck constraining countries’ efforts to improve their carbon neutrality policies, with 119 countries still not having such policies in place. While there is a relatively small gap between countries in terms of their fossil energy transition and deployment of zero-carbon technologies, the gap between their carbon neutrality technology innovation capacities and progress on climate investment and financing is wider. Although carbon neutrality technologies have been widely deployed across 178 countries, only 47 countries, mostly located in Europe and North America, possess innovation capabilities. An effective practice for financing pathways worldwide is still lacking, with 132 countries evidencing no progress in climate financing.

### 6.3 Global carbon neutrality effectiveness scores

Global carbon emissions are gradually being decoupled from economic growth, but the progress of decarbonization varies significantly across countries. Currently, 83 % of countries have reduced their carbon intensity, with 46 % achieving a reduction rate that is consistent with their carbon neutrality targets. In 12 countries, the peak carbon intensity has fallen by >75 %, and in 56 countries, the reduction exceeds 50 %. However, 51 countries have seen a reduction of <25 %. Some Asian and African developing countries are still experiencing rising carbon intensity and may potentially become future hotspots for carbon emissions.

## 7 Progress in nations’ carbon neutrality scores

**Fig. 9:**
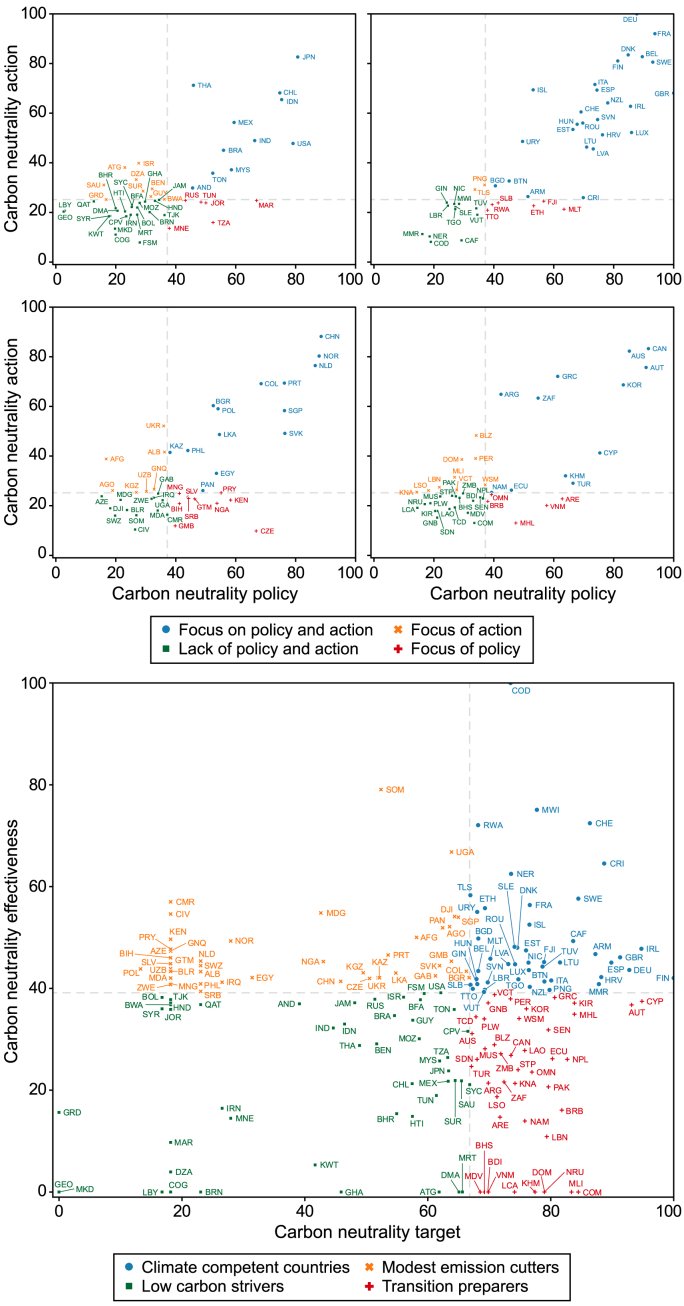
Classification of national carbon neutrality progress patterns in 2024 (the abbreviations are defined in Supplementary Material Table S3).

Countries' carbon-neutral ambitions and their effectiveness scores were not entirely correlated, and the decarbonization progress was not always consistent with targets. In the classification framework of this study, all countries were divided into four groups according to whether or not they ranked in the top 50 % of countries worldwide in terms of their ambition and effectiveness scores. These groups were: (1) ‘climate competent countries’, with scores in the top 50 % for both ambition and effectiveness; (2) ‘modest emission cutters’, with scores in the top 50 % and bottom 50 %, respectively, for effectiveness and ambition; (3) ‘transition preparers’, with scores in the top 50 % and bottom 50 %, respectively, for ambition and effectiveness; and ‘low carbon strivers’, with scores in the bottom 50 % for both ambition and effectiveness (Fig. 9).

During the current and previous years, 33 countries continued to lead the global process for becoming carbon neutral as ‘climate competent countries’. These countries generally belonged to three groups. The first comprised developed European countries, such as France, Germany, Denmark, and Finland, which have maintained their leading positions and actively participated in international climate cooperation through comprehensive support policies, technological innovations, and climate financing systems. The second comprised Eastern European countries such as Croatia, Romania, and Hungary, which achieved carbon peaking in the 1990s. These countries have largely decoupled economic development from emissions and have well-developed policy systems. The third group comprises emerging developing countries represented by Ethiopia, Costa Rica, the Central African Republic, and Rwanda, which have not only established carbon-neutral policy systems but have also actively developed carbon-neutral technologies. Some have performed outstandingly in the synergistic development of ecological protection and carbon-neutral targets. This year, there were 13 new ‘climate competent countries’, all of which were developing countries.

‘Transition preparers’ have changed considerably, with 28 countries remaining in the category. However, the countries that shifted were polarized: eight demonstrated increased effectiveness, transitioning to ‘climate competent countries’, and nine were demoted to ‘low carbon strivers’ because they showed reduced ambition. Developed countries continued to be ‘transition preparers’, generally demonstrating well-developed policy systems and advanced deployment of carbon-neutral technologies. However, their decarbonization progress has been hampered by slower fossil energy transition. In addition, their contributions to international cooperation are relatively low. Developing countries that remained in this category lacked common features and lagged behind in terms of their policies or actions, mainly because of insufficient financing support or lack of technology deployment.

The ‘modest emission cutters’ group remained relatively stable, comprising 34 countries. Despite being conservative in choosing the type, year, and coverage of their carbon-neutral targets, many developing countries demonstrated stellar performance relating to certain aspects of policy and action. For example, Bulgaria has introduced a number of corporate environmental, social and governance (ESG) disclosure policies and has many patents in areas such as digital industrial energy efficiency, CCUS, and electric vehicles, while countries such as El Salvador and Sri Lanka have set targets of 100 % electric vehicle sales. Moreover, some ‘low carbon strivers’ in 2023, who had achieved significant reductions in their carbon intensity, transitioned into ‘modest emission cutters’.

During 2023 and 2024, 32 countries remained ‘low carbon strivers’, demonstrating limited ambitions and effectiveness. These countries belonged to three groups. The first comprised oil-rich countries that are highly dependent on fossil fuels and are yet to decouple economic development from carbon emissions. The second comprised countries with large-scale economies and carbon emissions. Despite having abundant zero-carbon technology reserves or resource endowments as well as comprehensive policy systems, their actions have not effectively contributed to decarbonization. Examples include the United States, which is a global leader in the market deployment and innovation of biomass and CCS technologies; India, which has developed comprehensive carbon-neutral technologies and policy systems; and Brazil, which is at the forefront of biomass resources and technologies, globally. The third group comprises countries in the initial stages of economic development, with small-scale economies and carbon emission volumes. Most have not yet set carbon-neutral targets and have not initiated substantial carbon emission reduction processes.

In general, an increase in ambition was usually accompanied by improved policy support, while an increase in emission reduction effectiveness was mainly attributed to action-related progress. In 2024, 53 countries shifted in status. Nine regressed from ‘transition preparers’ to ‘low carbon strivers’ because of their reduced ambition, and 15 demonstrated increased effectiveness, with eight advancing from ‘transition preparers’ to ‘climate competent countries’ and seven advancing from ‘low carbon strivers’ to ‘modest emission cutters’. Lastly, eight countries demonstrating reduced effectiveness were demoted from ‘climate competent countries’ to ‘transition preparers’. Increases in national ambition were often accompanied by increases in scores for policy systems. Uruguay, for example, introduced a detailed roadmap for achieving carbon neutrality, along with several climate-related laws, well above the global average, and strategic renewable hydrogen targets. Improvements in national effectiveness mainly stemmed from action-related progress. For example, New Zealand introduced comprehensive corporate ESG disclosure policies, and Egypt has actively deployed renewable energy generation technologies, such as solar PV and onshore wind power, and has made significant progress in developing innovation relating to biofuels and electric vehicles.

## 8 Discussion and policy recommendations

Overall, although global commitments and actions aimed at achieving carbon neutrality continued to progress in 2024, substantial implementation gaps persisted. It is important to emphasize that bridging the implementation gap between targets and global progress toward carbon neutrality requires respecting the diversity of national carbon neutrality pathways.

Countries are at different stages of development, and developing countries need to achieve a faster pace of carbon reduction. Among the 49 countries that have already reached their carbon peak, the average interval between their peaking and carbon neutrality target is 49 years. Of these countries, 31 are developed countries with an average interval of 48 years. By contrast, for countries that have not yet peaked—over 99 % of which are developing countries—the average interval between the carbon peak and neutrality targets is just 21 years. This means that developing countries will need to reduce carbon emissions more rapidly after reaching their peak, accelerating the decoupling of their economies from energy consumption and carbon emissions. To achieve the shared global goal of emissions reduction, it is essential that differences in the developmental stages of countries that have already peaked and those that are yet to do so are fully respected.

A single zero-carbon pathway cannot accommodate differences in resource endowments, development stages, legal systems, and technological approaches across countries. Leveraging their advanced technological innovation and economic levels, developed countries are advantaged in areas such as plug-in hybrid vehicles, biodiesel, and CCS. Developing countries are, however, better positioned to utilize technologies such as hydropower, bioethanol, and forest carbon sinks, while relying on local resources. When selecting technologies, large countries, such as the United States, China, and Canada, have pursued multi-technology support policies, while European countries have focused on advances in transportation, for example, electric vehicles and renewable hydrogen. Countries in Southeast Asia and Africa have favoured biofuels and carbon sink technologies. Policy approaches also vary: both China and the United States have implemented a mix of mandatory and incentive-based policies, whereas European countries have relied predominantly on incentives.

Differentiated strategies for achieving carbon neutrality are needed for different groups of countries. ‘Climate competent countries’ should maintain their technological and policy leadership and export their experience and technology through international cooperation. ‘Transition preparers’ should accelerate their actions and policy implementation, especially in the areas of fossil fuel transformation and technological innovation. ‘Modest emission cutters’ should increase their carbon neutrality ambitions and policy support, while ‘low carbon strivers’ should promptly establish decarbonization pathways, strengthen their technology adoption and policy frameworks, and seek international financial and technical support to advance low-carbon development.

To accelerate global carbon neutrality, urgent action is needed in the areas of finance, technology, and international cooperation, especially for supporting developing countries. Strengthening information disclosure and the assessment of technological and financial needs to promote effective global diffusion of carbon emission reduction technologies and climate finance is a shared requirement for accelerating the global process for achieving carbon neutrality. Currently, the disclosure of technology and finance data is inadequate, especially on market penetration of cutting-edge, carbon-neutral technologies and the transparency of climate finance. This impedes the provision of clear guidelines for investors and the inflow of funds and technologies. In particular, the lack of public data on financial flows and needs assessments traps developing countries, in an ‘opaque information—unclear needs—insufficient assistance’ cycle. To ensure a just transition to global carbon neutrality and bridge the gap between goals and outcomes, it is imperative to improve data collection and integration on carbon-neutral technologies, investments, financing, and international cooperation. Building a transparent and credible global database and evaluation system and enhancing public governance infrastructure are essential for accelerating the achievement of global carbon neutrality.

## 9 Conclusions

In light of rising, record-breaking temperatures and increasingly frequent extreme weather events, addressing climate change and achieving carbon neutrality have become shared global objectives. We developed a comprehensive ‘target–policy–action–effectiveness’ indicator system to track the progress of global carbon neutrality, assess the implementation gap between goals and actions, and identify opportunities and challenges faced by various countries. These efforts are essential for accelerating both global and national climate governance, facilitating equitable transitions, and advancing sustainable development.

The process of achieving global carbon neutrality has now transitioned from goal setting to implementation. From June 2023 to May 2024, the number of countries with carbon neutrality targets increased from 133 to 151, of which 120 have established the legal status of their targets. Zero-carbon technologies have been rapidly deployed worldwide. Globally, new renewable energy installations, light-duty electric vehicle sales, and numbers of green hydrogen and CCUS projects increased by 14 %, 30 %, 40 %, and 110 %, respectively. Moreover, developed countries’ commitments to climate finance have increased. Developed countries were projected to provide USD 94.7 billion annually for international climate finance by 2025, but the actual amount remains below the USD 100 billion contribution target. Overall, the gap between leading nations and other countries has narrowed, with many countries demonstrating substantial progress in setting carbon neutrality targets. Spurred by new commitments from developing countries and broader target coverage in developed nations, the global median target score increased from 61 in 2023 to 65.

However, there remains a significant implementation bottleneck that constrains the achievement of the global carbon neutrality target. Currently, only 72 countries have established comprehensive carbon neutrality policy frameworks. Far from tripling the renewable energy target, if the current growth trend continues, the installed capacity of renewable energy will only reach a maximum of 2.7 times the 2022 level by 2030. The policy ambition and planned capacity for several zero-carbon technologies require substantial enhancement to meet the climate targets. Moreover, there is a significant gap between the global need for climate investment and finance and actual investment levels. Achieving the 2 °C target requires a three to fivefold increase in global climate investments in mitigation, with an even greater need in least developed countries, which require a four to sevenfold increase. While international cooperation on carbon neutrality has expanded, substantial gaps remain in the quality of collaboration and financial implementation. In some cases, policies enacted by certain countries have even obstructed the effective global spread of carbon-neutral technologies.

Narrowing the implementation gap is a shared global imperative. Currently, the global median action score is just 25, which is well below the target score of 65. Improving information disclosure and assessing technological and financial needs are essential steps for facilitating the effective global spread of carbon reduction technologies and climate finance, both of which are crucial for accelerating progress on the global path to carbon neutrality. Additionally, a single zero-carbon pathway cannot account for the diverse resource endowments, development stages, legal systems, and technological approaches in different countries. Bridging the implementation gap requires full acknowledgement and support for the diverse pathways that nations may pursue towards carbon neutrality.

Although some aspects of our analysis are somewhat superficial, we have nevertheless provided comprehensive and systematic insights into global progress towards carbon neutrality. Future studies could focus on expanding the analytical dimensions by assessing the social, economic, and climate impacts of carbon neutrality actions and establishing more comprehensive and transparent databases, for example, through the incorporation of city-level and mining policy data.

## CRediT authorship contribution statement

**Shihui Zhang:** Writing – review & editing, Writing – original draft, Conceptualization. **Wenjia Cai:** Conceptualization, Writing – review & editing, Writing – original draft. **Xinzhu Zheng:** Writing – review & editing, Conceptualization, Writing – original draft. **Xuedu Lv:** Writing – review & editing. **Kangxin An:** Visualization, Data curation, Formal analysis. **Yuan Cao:** Data curation, Formal analysis, Visualization. **Hou Sang Cheng:** Visualization, Data curation, Formal analysis. **Jingyi Dai:** Visualization, Data curation, Formal analysis. **Xinyang Dong:** Visualization, Data curation, Formal analysis. **Shuting Fan:** Visualization, Data curation, Formal analysis. **Yiying Gao:** Visualization, Data curation, Formal analysis. **Zaizuo Gong:** Visualization, Data curation, Formal analysis. **Yusheng Guan:** Visualization, Data curation, Formal analysis. **Congkai Hong:** Visualization, Data curation, Formal analysis. **Jie Li:** Visualization, Data curation, Formal analysis. **Mingyu Li:** Visualization, Data curation, Formal analysis. **Yukai Li:** Visualization, Data curation, Formal analysis. **Songrun Liang:** Data curation, Formal analysis, Visualization. **Weiyi Liao:** Visualization, Data curation, Formal analysis. **Zhongqi Ma:** Data curation, Formal analysis, Visualization. **Yue Ren:** Visualization, Data curation, Formal analysis. **Jianxiang Shen:** Writing – review & editing, Writing – original draft, Data curation, Formal analysis, Visualization. **Xin Shi:** Data curation, Formal analysis, Visualization. **Yang Su:** Visualization, Data curation, Formal analysis. **Jinjie Sun:** Visualization, Data curation, Formal analysis. **Chengqi Xia:** Visualization, Data curation, Formal analysis. **Junyuan Xu:** Visualization, Data curation, Formal analysis. **Wenxin Ye:** Visualization, Data curation, Formal analysis. **Ling Zhang:** Visualization, Data curation, Formal analysis. **Shangchen Zhang:** Visualization, Data curation, Formal analysis. **Tianyi Zhang:** Visualization, Data curation, Formal analysis. **Zihan Zhen:** Writing – review & editing, Writing – original draft, Visualization, Data curation, Formal analysis. **Hanying Zhong:** Visualization, Data curation, Formal analysis. **Can Wang:** Conceptualization. **Kebin He:** Conceptualization.

## Declaration of competing interest

The authors declare that they have no known competing financial interests or personal relationships that could have appeared to influence the work reported in this paper.

The authors declare the following financial interests/personal relationships which may be considered as potential competing interests: Shihui Zhang, Wenjia Cai, Xinzhu Zheng, Xuedu Lv, Kangxin An, Yuan Cao, Hou Sang Cheng, Jingyi Dai, Xinyang Dong, Shuting Fan, Yiying Gao, Zaizuo Gong, Yusheng Guan, Congkai Hong, Jie Li, Mingyu Li, Yukai Li, Songrun Liang, Weiyi Liao, Zhongqi Ma, Yue Ren, Jianxiang Shen, Xin Shi, Yang Su, Jinjie Sun, Chengqi Xia, Junyuan Xu, Wenxin Ye, Ling Zhang, Shangchen Zhang, Tianyi Zhang, Zihan Zhen, Hanying Zhong, Can Wang, Kebin He.
