# Supplementary material for: Global readiness for carbon neutrality: From targets to action
Source: Environ Sci Ecotechnol. 2025 Mar 7;25:100546. doi: 10.1016/j.ese.2025.100546 (PMC11951988; doi:10.1016/j.ese.2025.100546)
Supplement: Multimedia component 1 [file mmc1.docx]

## Appendix A. Supplementary Information

Table. S1. Indicators System for the 2024 Global Carbon Neutrality Annual Progress Report

| **special topic** | **Level 1 indicators** | **Secondary indicators** | **Tertiary indicators** |
| --- | --- | --- | --- |
| Target | 1.1 Carbon neutral/Net zero emissions targets | 1.1.1 Types of carbon neutral targets | Target type |
| Target | 1.1 Carbon neutral/Net zero emissions targets | 1.1.2 Carbon neutral target year | Target year |
| Target | 1.2 Coverage of carbon neutral targets | 1.2.1 Sectoral coverage of carbon neutral targets | Coverage of international aviation |
| Target | 1.2 Coverage of carbon neutral targets | 1.2.1 Sectoral coverage of carbon neutral targets | Coverage of international shipping |
| Target | 1.2 Coverage of carbon neutral targets | 1.2.2 Greenhouse gas coverage of carbon neutral targets | Greenhouse gas coverage |
| Target | 1.2 Coverage of carbon neutral targets | 1.2.3 Consumption emissions coverage of carbon neutral targets | Coverage of consumption emissions |
| Target | 1.2 Coverage of carbon neutral targets | 1.2.4 Territorial emissions coverage of in carbon neutral targets | Coverage of all territorial emissions |
| Target | 1.2 Coverage of carbon neutral targets | 1.2.5 Validity and reliability of carbon neutral targets | Independent carbon removal targets |
| Target | 1.2 Coverage of carbon neutral targets | 1.2.5 Validity and reliability of carbon neutral targets | Planned use of carbon credits |
| Target | 1.3 Carbon neutral target regulatory system | 1.3.1 Legal completeness of carbon neutral targets | Status of target legislation |
| Target | 1.3 Carbon neutral target regulatory system | 1.3.2 Regulatory mechanisms for carbon neutral targets | Review and reporting system |
| Target | 1.3 Carbon neutral target regulatory system | 1.3.2 Regulatory mechanisms for carbon neutral targets | Accountability system |
| Target | 1.3 Carbon neutral target regulatory system | 1.3.3 Legal action on carbon neutral targets | Number of climate change laws |
| Target | 1.3 Carbon neutral target regulatory system | 1.3.3 Legal action on carbon neutral targets | Number of climate change litigation cases |
| Target | 1.4 Specific roadmap for carbon neutral targets | 1.4.1 Milestones for carbon neutral targets | Short- and medium-term targets |
| Target | 1.4 Specific roadmap for carbon neutral targets | 1.4.1 Milestones for carbon neutral targets | Texts of short- and medium-term targets |
| Target | 1.4 Specific roadmap for carbon neutral targets | 1.4.1 Milestones for carbon neutral targets | Years of short- and medium-term targets |
| Target | 1.4 Specific roadmap for carbon neutral targets | 1.4.1 Milestones for carbon neutral targets | Types of short- and medium-term targets |
| Target | 1.4 Specific roadmap for carbon neutral targets | 1.4.2 National carbon neutral roadmap | Detailed plans |
| Target | 1.4 Specific roadmap for carbon neutral targets | 1.4.2 National carbon neutral roadmap | Whether to outline the plan |
| Target | 1.4 Specific roadmap for carbon neutral targets | 1.4.3 Carbon neutrality at the region level | Percentage of regions with policies |
| Target | 1.4 Specific roadmap for carbon neutral targets | 1.4.3 Carbon neutrality at the region level | Percentage of cities with policies |
| Target | 1.4 Specific roadmap for carbon neutral targets | 1.4.4 Carbon neutrality at the industry level | Energy supply |
| Target | 1.4 Specific roadmap for carbon neutral targets | 1.4.4 Carbon neutrality at the industry level | Agriculture |
| Target | 1.4 Specific roadmap for carbon neutral targets | 1.4.4 Carbon neutrality at the industry level | Transportation |
| Target | 1.4 Specific roadmap for carbon neutral targets | 1.4.4 Carbon neutrality at the industry level | Land use and forestry |
| Target | 1.4 Specific roadmap for carbon neutral targets | 1.4.4 Carbon neutrality at the industry level | Construction industry |
| Target | 1.4 Specific roadmap for carbon neutral targets | 1.4.4 Carbon neutrality at the industry level | Wastes industry |
| Target | 1.4 Specific roadmap for carbon neutral targets | 1.4.4 Carbon neutrality at the industry level | Industries |
| Target | 1.5 Equity and coherence of carbon neutral targets | 1.5.1 Equity of carbon neutral targets | Reference to equity |
| Target | 1.5 Equity and coherence of carbon neutral targets | 1.5.1 Equity of carbon neutral targets | Clear explanation on equity |
| Target | 1.5 Equity and coherence of carbon neutral targets | 1.5.2 Alignment of carbon neutral targets with global targets | Meet the 1.5 °C target |
| Target | 1.5 Equity and coherence of carbon neutral targets | 1.5.2 Alignment of carbon neutral targets with global targets | Meet the 2 °C target |
| Target | 1.5 Equity and coherence of carbon neutral targets | 1.5.2 Alignment of carbon neutral targets with global targets | Carbon neutral target ambition index at the 1.5 °C Target |
| Technology | 2.1 Renewable energy generation technologies | 2.1.1 Strategic targets for renewable energy generation | Targets on renewable energy share/fossil fuel share in future electricity generation |
| Technology | 2.1 Renewable energy generation technologies | 2.1.1 Strategic targets for renewable energy generation | Targets on renewable hydrogen by the end of 2022 |
| Technology | 2.1 Renewable energy generation technologies | 2.1.1 Strategic targets for renewable energy generation | Targets proposed on future share of renewable energy in electricity generation by the end of 2022 |
| Technology | 2.1 Renewable energy generation technologies | 2.1.2 Support policies for renewable energy generation | Fossil fuel ban |
| Technology | 2.1 Renewable energy generation technologies | 2.1.2 Support policies for renewable energy generation | Renewable energy policies |
| Technology | 2.1 Renewable energy generation technologies | 2.1.2 Support policies for renewable energy generation | Net-zero emissions policy |
| Technology | 2.1 Renewable energy generation technologies | 2.1.2 Support policies for renewable energy generation | Electric utility quota obligation/RPS |
| Technology | 2.1 Renewable energy generation technologies | 2.1.2 Support policies for renewable energy generation | Distributed renewable energy in INDC & NDC |
| Technology | 2.1 Renewable energy generation technologies | 2.1.2 Support policies for renewable energy generation | Net metering/billing policy |
| Technology | 2.1 Renewable energy generation technologies | 2.1.2 Support policies for renewable energy generation | Feed-in tariff/premium payment policy |
| Technology | 2.1 Renewable energy generation technologies | 2.1.2 Support policies for renewable energy generation | Biofuel blend/Renewable transportation policy |
| Technology | 2.1 Renewable energy generation technologies | 2.1.2 Support policies for renewable energy generation | Heat supply related policies |
| Technology | 2.1 Renewable energy generation technologies | 2.1.2 Support policies for renewable energy generation | Tradable REC |
| Technology | 2.1 Renewable energy generation technologies | 2.1.2 Support policies for renewable energy generation | Distributed renewable energy |
| Technology | 2.1 Renewable energy generation technologies | 2.1.2 Support policies for renewable energy generation | Distributed renewable energy integration |
| Technology | 2.1 Renewable energy generation technologies | 2.1.2 Support policies for renewable energy generation | Administrative and legal provisions |
| Technology | 2.1 Renewable energy generation technologies | 2.1.2 Support policies for renewable energy generation | Bidding policy |
| Technology | 2.1 Renewable energy generation technologies | 2.1.2 Support policies for renewable energy generation | Quality/technology frameworks and standards |
| Technology | 2.1 Renewable energy generation technologies | 2.1.2 Support policies for renewable energy generation | Public financing |
| Technology | 2.1 Renewable energy generation technologies | 2.1.2 Support policies for renewable energy generation | Financial incentives |
| Technology | 2.1 Renewable energy generation technologies | 2.1.2 Support policies for renewable energy generation | Tax reduction policy |
| Technology | 2.1 Renewable energy generation technologies | 2.1.2 Support policies for renewable energy generation | Tax credit for investment or production |
| Technology | 2.1 Renewable energy generation technologies | 2.1.2 Support policies for renewable energy generation | Investment subsidies related policies |
| Technology | 2.1 Renewable energy generation technologies | 2.1.3 Progress in renewable energy generation | Renewable hydropower generation GWh |
| Technology | 2.1 Renewable energy generation technologies | 2.1.3 Progress in renewable energy generation | Photovoltaic power generation GWh |
| Technology | 2.1 Renewable energy generation technologies | 2.1.3 Progress in renewable energy generation | Onshore wind power generation GWh |
| Technology | 2.1 Renewable energy generation technologies | 2.1.3 Progress in renewable energy generation | Geothermal energy power generation GWh |
| Technology | 2.1 Renewable energy generation technologies | 2.1.3 Progress in renewable energy generation | Renewable waste power generation GWh |
| Technology | 2.1 Renewable energy generation technologies | 2.1.3 Progress in renewable energy generation | Renewable hydropower installed capacity MW |
| Technology | 2.1 Renewable energy generation technologies | 2.1.3 Progress in renewable energy generation | Photovoltaic power installed capacity MW |
| Technology | 2.1 Renewable energy generation technologies | 2.1.3 Progress in renewable energy generation | Onshore wind power installed capacity MW |
| Technology | 2.1 Renewable energy generation technologies | 2.1.3 Progress in renewable energy generation | Renewable waste power installed capacity MW |
| Technology | 2.1 Renewable energy generation technologies | 2.1.3 Progress in renewable energy generation | Geothermal power installed capacity MW |
| Technology | 2.1 Renewable energy generation technologies | 2.1.4 Innovation in renewable energy generation technologies | Levelized cost of energy for onshore wind power generation |
| Technology | 2.1 Renewable energy generation technologies | 2.1.4 Innovation in renewable energy generation technologies | Levelized cost of energy for solar power generation |
| Technology | 2.1 Renewable energy generation technologies | 2.1.4 Innovation in renewable energy generation technologies | Levelized cost of energy for offshore wind power generation |
| Technology | 2.1 Renewable energy generation technologies | 2.1.4 Innovation in renewable energy generation technologies | Levelized cost of energy for hydropower generation |
| Technology | 2.1 Renewable energy generation technologies | 2.1.4 Innovation in renewable energy generation technologies | Levelized cost of energy for biomass power generation |
| Technology | 2.2 Electric vehicles | 2.2.1 Strategic targets for electric vehicles | Targets on electric vehicle sales share |
| Technology | 2.2 Electric vehicles | 2.2.1 Strategic targets for electric vehicles | Targets on electric vehicle stock |
| Technology | 2.2 Electric vehicles | 2.2.1 Strategic targets for electric vehicles | Targets on electric vehicle stock share |
| Technology | 2.2 Electric vehicles | 2.2.2 Support policies for electric vehicle | Command management |
| Technology | 2.2 Electric vehicles | 2.2.2 Support policies for electric vehicle | Subsidies |
| Technology | 2.2 Electric vehicles | 2.2.2 Support policies for electric vehicle | Tax credits |
| Technology | 2.2 Electric vehicles | 2.2.2 Support policies for electric vehicle | Complementary measures |
| Technology | 2.2 Electric vehicles | 2.2.3 Progress in electric vehicle | Plug-in hybrid vehicle stock |
| Technology | 2.2 Electric vehicles | 2.2.3 Progress in electric vehicle | Plug-in hybrid vehicle sales |
| Technology | 2.2 Electric vehicles | 2.2.3 Progress in electric vehicle | Battery-powered vehicle stock |
| Technology | 2.2 Electric vehicles | 2.2.3 Progress in electric vehicle | Battery-powered vehicle sales |
| Technology | 2.2 Electric vehicles | 2.2.3 Progress in electric vehicle | Electric vehicle stock share |
| Technology | 2.2 Electric vehicles | 2.2.3 Progress in electric vehicle | Electric vehicle sales share |
| Technology | 2.2 Electric vehicles | 2.2.3 Progress in electric vehicle | Fuel cell vehicle sales |
| Technology | 2.2 Electric vehicles | 2.2.3 Progress in electric vehicle | Fuel cell vehicle stock |
| Technology | 2.2 Electric vehicles | 2.2.4 Innovation in electric vehicle | Number of patents |
| Technology | 2.2 Electric vehicles | 2.2.4 Innovation in electric vehicle | Number of papers |
| Technology | 2.3 Energy efficiency | 2.3.1 Strategic targets for energy efficiency | Overall energy efficiency phase targets such as in 2025/2030 |
| Technology | 2.3 Energy efficiency | 2.3.1 Strategic targets for energy efficiency | Staged energy efficiency phase targets for industry,construction, transportation, etc. |
| Technology | 2.3 Energy efficiency | 2.3.2 Support policies for energy efficiency | Support policies |
| Technology | 2.3 Energy efficiency | 2.3.3 Progress in energy efficiency | Share of sales of low-emission vehicles |
| Technology | 2.3 Energy efficiency | 2.3.3 Progress in energy efficiency | Heat pump market share |
| Technology | 2.3 Energy efficiency | 2.3.3 Progress in energy efficiency | Solar water heater installed area per capita |
| Technology | 2.3 Energy efficiency | 2.3.4 Innovation in energy efficiency | Number of patent applications for industrial digitization |
| Technology | 2.3 Energy efficiency | 2.3.4 Innovation in energy efficiency | Number of patent applications for heat pump technology |
| Technology | 2.3 Energy efficiency | 2.3.4 Innovation in energy efficiency | Number of patent applications for low-emission vehicles |
| Technology | 2.3 Energy efficiency | 2.3.4 Innovation in energy efficiency | Number of patents |
| Technology | 2.4 Biofuels | 2.4.1 Strategic targets for biofuels | Target year for fuel ethanol blending |
| Technology | 2.4 Biofuels | 2.4.1 Strategic targets for biofuels | Fuel ethanol blending targets |
| Technology | 2.4 Biofuels | 2.4.1 Strategic targets for biofuels | Target year for biodiesel blending |
| Technology | 2.4 Biofuels | 2.4.1 Strategic targets for biofuels | Biodiesel blending targets |
| Technology | 2.4 Biofuels | 2.4.2 Support policies for biofuels | Biodiesel mandatory ratio |
| Technology | 2.4 Biofuels | 2.4.2 Support policies for biofuels | Fuel ethanol mandatory ratio |
| Technology | 2.4 Biofuels | 2.4.3 Progress in biofuels | Fuel ethanol production |
| Technology | 2.4 Biofuels | 2.4.3 Progress in biofuels | Biodiesel consumption |
| Technology | 2.4 Biofuels | 2.4.3 Progress in biofuels | Biodiesel production |
| Technology | 2.4 Biofuels | 2.4.3 Progress in biofuels | Fuel ethanol consumption |
| Technology | 2.4 Biofuels | 2.4.4 Innovation in biofuels | Number of papers |
| Technology | 2.4 Biofuels | 2.4.4 Innovation in biofuels | Number of patents |
| Technology | 2.5 CCUS | 2.5.1 Strategic targets for CCUS | Technology positioning/development targets in policy documents |
| Technology | 2.5 CCUS | 2.5.2 Support policies for CCUS | R&D support |
| Technology | 2.5 CCUS | 2.5.2 Support policies for CCUS | Pilot project support |
| Technology | 2.5 CCUS | 2.5.2 Support policies for CCUS | Government funding support |
| Technology | 2.5 CCUS | 2.5.2 Support policies for CCUS | Tax credits/carbon markets |
| Technology | 2.5 CCUS | 2.5.2 Support policies for CCUS | Incorporating Nationally Determined Contributions under the Paris Agreement |
| Technology | 2.5 CCUS | 2.5.2 Support policies for CCUS | Whether to join the Carbon Management Challenge (CMC) |
| Technology | 2.5 CCUS | 2.5.2 Support policies for CCUS | Construction of the regulatory system |
| Technology | 2.5 CCUS | 2.5.3 Progress in CCUS | Number of CCUS projects in operation |
| Technology | 2.5 CCUS | 2.5.3 Progress in CCUS | Number of projects planned |
| Technology | 2.5 CCUS | 2.5.4 Innovation in CCUS | Number of patents |
| Technology | 2.5 CCUS | 2.5.4 Innovation in CCUS | Number of papers |
| Technology | 2.6 Renewable hydrogen | 2.6.1 Strategic targets for renewable hydrogen | Target year for installation of electrolytic tanks |
| Technology | 2.6 Renewable hydrogen | 2.6.1 Strategic targets for renewable hydrogen | Electrolytic tanks installation target |
| Technology | 2.6 Renewable hydrogen | 2.6.2 Support policies for renewable hydrogen | Subsidizing cleaner hydrogen production |
| Technology | 2.6 Renewable hydrogen | 2.6.2 Support policies for renewable hydrogen | Encouraging the production of hydrogen-based derivatives |
| Technology | 2.6 Renewable hydrogen | 2.6.2 Support policies for renewable hydrogen | Promoting hydrogen infrastructure |
| Technology | 2.6 Renewable hydrogen | 2.6.2 Support policies for renewable hydrogen | Promote investment in R&D of hydrogen energy technologies |
| Technology | 2.6 Renewable hydrogen | 2.6.2 Support policies for renewable hydrogen | Promoting international cooperation on hydrogen energy |
| Technology | 2.6 Renewable hydrogen | 2.6.2 Support policies for renewable hydrogen | Promoting hydrogen energy regulation and standard system |
| Technology | 2.6 Renewable hydrogen | 2.6.3 Progress in renewable hydrogen | Green hydrogen production capacity |
| Technology | 2.6 Renewable hydrogen | 2.6.3 Progress in renewable hydrogen | Number of green hydrogen projects commissioned |
| Technology | 2.6 Renewable hydrogen | 2.6.4 Innovation in renewable hydrogen | Number of patents |
| Technology | 2.6 Renewable hydrogen | 2.6.4 Innovation in renewable hydrogen | Number of papers |
| Technology | 2.7 Carbon sink | 2.7.1 Strategic targets for carbon sink | Global forest goal 1-6 |
| Technology | 2.7 Carbon sink | 2.7.2 Support policies for carbon sink | Participation in REDD+ actions; average annual carbon emissions reduction; amount of support funds received |
| Technology | 2.7 Carbon sink | 2.7.2 Support policies for carbon sink | Contribution of national LULUCF credits to carbon emission reductions |
| Technology | 2.7 Carbon sink | 2.7.2 Support policies for carbon sink | Availability of voluntary national reporting in response to the United Nations Strategic Plan for Forests 2030; identification of national contributions to the achievement of the global objectives on forests |
| Technology | 2.7 Carbon sink | 2.7.3 Progress in carbon sink | Afforestation and reforestation CDM project; Agriculture CDM project |
| Technology | 2.7 Carbon sink | 2.7.3 Progress in carbon sink | Forest carbon stock amount |
| Technology | 2.7 Carbon sink | 2.7.4 Innovation in carbon sink | Number of patents |
| Technology | 2.7 Carbon sink | 2.7.4 Innovation in carbon sink | Number of papers |
| Finance | 3.1 Climate investment and finance support policies | 3.1.1 Pledge of commitment by countries to climate investment and finance | Whether the national LT-LEDS refer to climate investment and finance |
| Finance | 3.1 Climate investment and finance support policies | 3.1.1 Pledge of commitment by countries to climate investment and finance | Whether national NDCs refer to climate investment and finance |
| Finance | 3.1 Climate investment and finance support policies | 3.1.1 Pledge of commitment by countries to climate investment and finance | Latest NDC release time for each country |
| Finance | 3.1 Climate investment and finance support policies | 3.1.1 Pledge of commitment by countries to climate investment and finance | Latest release time for national LT-LEDS |
| Finance | 3.1 Climate investment and finance support policies | 3.1.2 Climate investment and finance support policies | Number of sustainable financial policies |
| Finance | 3.1 Climate investment and finance support policies | 3.1.3 National assessment and disclosure of climate-related financial risks | Whether a climate-related financial risk assessment has been carried out |
| Finance | 3.1 Climate investment and finance support policies | 3.1.3 National assessment and disclosure of climate-related financial risks | Whether disclosure of climate-related financial risks is mandatory |
| Finance | 3.2 Progress on action on climate finance and investment | 3.2.1 Proportion of financial budget for climate-related actions | Scale of national budgets for mitigation actions |
| Finance | 3.2 Progress on action on climate finance and investment | 3.2.1 Proportion of financial budget for climate-related actions | Scale of national financial budgets for adaptation actions |
| Finance | 3.2 Progress on action on climate finance and investment | 3.2.2 National carbon pricing mechanisms | Progress on national carbon pricing mechanisms |
| Finance | 3.2 Progress on action on climate finance and investment | 3.2.3 Developed countries’ climate finance commitments and implementation of international climate finance | Developed country commitments for international climate finance |
| Finance | 3.2 Progress on action on climate finance and investment | 3.2.3 Developed countries’ climate finance commitments and implementation of international climate finance | Loss and damage funding commitments |
| Finance | 3.2 Progress on action on climate finance and investment | 3.2.4 Climate finance contributions in the GEF and GCF | National contributions to the Green Climate Fund under the Convention (GCF-1) |
| Finance | 3.2 Progress on action on climate finance and investment | 3.2.4 Climate finance contributions in the GEF and GCF | National contributions to the Green Climate Fund under the Convention (GCF-2) |
| Finance | 3.2 Progress on action on climate finance and investment | 3.2.4 Climate finance contributions in the GEF and GCF | Contributions to the Green Climate Fund by countries under the Convention (GEF) |
| Finance | 3.2 Progress on action on climate finance and investment | 3.2.5 Scale of funding communicated in the biennial reports | Scale of funding communicated by developed countries in their biennial reports (BRs) |
| Finance | 3.2 Progress on action on climate finance and investment | 3.2.5 Scale of funding communicated in the biennial reports | Scale of funding received as communicated by developing countries in their biennial update reports (BURs) |
| Finance | 3.2 Progress on action on climate finance and investment | 3.2.6 Participation in international climate finance cooperation initiatives | Number of institutions in each country that are members of GFANZ |
| Finance | 3.2 Progress on action on climate finance and investment | 3.2.6 Participation in international climate finance cooperation initiatives | Number of institutions in each country that have signed the UN PRI |
| International Cooperation | 4.1 International technology cooperation | 4.1.1 International technology transfer projects | Field |
| International Cooperation | 4.1 International technology cooperation | 4.1.1 International technology transfer projects | Support type |
| International Cooperation | 4.1 International technology cooperation | 4.1.1 International technology transfer projects | Category |
| International Cooperation | 4.1 International technology cooperation | 4.1.1 International technology transfer projects | Keywords |
| International Cooperation | 4.1 International technology cooperation | 4.1.1 International technology transfer projects | Host country |
| International Cooperation | 4.1 International technology cooperation | 4.1.1 International technology transfer projects | Recipient country |
| International Cooperation | 4.1 International technology cooperation | 4.1.1 International technology transfer projects | Start time |
| International Cooperation | 4.1 International technology cooperation | 4.1.1 International technology transfer projects | End time |
| International Cooperation | 4.2 Obstacles to international cooperation | 4.2.1 Green trade barriers | Number of trade barriers |

Table. S2. Standardized 0-100 scoring criteria for national carbon neutrality progress indicators

| **Indicator type** | **Score** | **Score description and examples** |
| --- | --- | --- |
| Target | 100 | Optimal situation: e.g. carbon neutrality commitment to cover all GHGs; |
|  | 0 | No carbon neutrality commitment |
| Policy | 100 | Current best practice: e.g. comprehensive policies supporting all types of carbon neutrality technologies; established systems for climate-related financial risk assessment and disclosure |
|  | 0 | No carbon neutrality policy |
| Action | 100 | Current best practice: e.g. highest worldwide electric vehicle ownership per unit of carbon emissions; highest number of CCUS projects per unit of carbon emissions; highest issuance of green bonds per capita |
|  | 0 | No carbon neutrality action |
| Effectiveness | 100 | Best practice adjusted for reduction difficulty: e.g., countries with historically high carbon intensity (up to three times the global average) have achieved over 80% reduction; annual rate of carbon intensity reduction aligns with carbon neutrality targets |
|  | 0 | No change or increase in carbon intensity |

Table. S3. ISO abbreviations of countries and regions

| **Country or Area** | **ISO** | **Country or Area** | **ISO** |
| --- | --- | --- | --- |
| Afghanistan | AFG | Latvia | LVA |
| Åland Islands | ALA | Lebanon | LBN |
| Albania | ALB | Lesotho | LSO |
| Algeria | DZA | Liberia | LBR |
| American Samoa | ASM | Libya | LBY |
| Andorra | AND | Liechtenstein | LIE |
| Angola | AGO | Lithuania | LTU |
| Anguilla | AIA | Luxembourg | LUX |
| Antarctica | ATA | Madagascar | MDG |
| Antigua and Barbuda | ATG | Malawi | MWI |
| Argentina | ARG | Malaysia | MYS |
| Armenia | ARM | Maldives | MDV |
| Aruba | ABW | Mali | MLI |
| Australia | AUS | Malta | MLT |
| Austria | AUT | Marshall Islands | MHL |
| Azerbaijan | AZE | Martinique | MTQ |
| Bahamas | BHS | Mauritania | MRT |
| Bahrain | BHR | Mauritius | MUS |
| Bangladesh | BGD | Mayotte | MYT |
| Barbados | BRB | Mexico | MEX |
| Belarus | BLR | Micronesia (Federated States of) | FSM |
| Belgium | BEL | Monaco | MCO |
| Belize | BLZ | Mongolia | MNG |
| Benin | BEN | Montenegro | MNE |
| Bermuda | BMU | Montserrat | MSR |
| Bhutan | BTN | Morocco | MAR |
| Bolivia (Plurinational State of) | BOL | Mozambique | MOZ |
| Bonaire, Sint Eustatius and Saba | BES | Myanmar | MMR |
| Bosnia and Herzegovina | BIH | Namibia | NAM |
| Botswana | BWA | Nauru | NRU |
| Bouvet Island | BVT | Nepal | NPL |
| Brazil | BRA | Netherlands (Kingdom of the) | NLD |
| British Indian Ocean Territory | IOT | New Caledonia | NCL |
| British Virgin Islands | VGB | New Zealand | NZL |
| Brunei Darussalam | BRN | Nicaragua | NIC |
| Bulgaria | BGR | Niger | NER |
| Burkina Faso | BFA | Nigeria | NGA |
| Burundi | BDI | Niue | NIU |
| Cabo Verde | CPV | Norfolk Island | NFK |
| Cambodia | KHM | North Macedonia | MKD |
| Cameroon | CMR | Northern Mariana Islands | MNP |
| Canada | CAN | Norway | NOR |
| Cayman Islands | CYM | Oman | OMN |
| Central African Republic | CAF | Pakistan | PAK |
| Chad | TCD | Palau | PLW |
| Chile | CHL | Panama | PAN |
| China | CHN | Papua New Guinea | PNG |
| China, Hong Kong Special Administrative Region | HKG | Paraguay | PRY |
| China, Macao Special Administrative Region | MAC | Peru | PER |
| Christmas Island | CXR | Philippines | PHL |
| Cocos (Keeling) Islands | CCK | Pitcairn | PCN |
| Colombia | COL | Poland | POL |
| Comoros | COM | Portugal | PRT |
| Congo | COG | Puerto Rico | PRI |
| Cook Islands | COK | Qatar | QAT |
| Costa Rica | CRI | Republic of Korea | KOR |
| Côte d’Ivoire | CIV | Republic of Moldova | MDA |
| Croatia | HRV | Réunion | REU |
| Cuba | CUB | Romania | ROU |
| Curaçao | CUW | Russian Federation | RUS |
| Cyprus | CYP | Rwanda | RWA |
| Czechia | CZE | Saint Barthélemy | BLM |
| Democratic People's Republic of Korea | PRK | Saint Helena | SHN |
| Democratic Republic of the Congo | COD | Saint Kitts and Nevis | KNA |
| Denmark | DNK | Saint Lucia | LCA |
| Djibouti | DJI | Saint Martin (French Part) | MAF |
| Dominica | DMA | Saint Pierre and Miquelon | SPM |
| Dominican Republic | DOM | Saint Vincent and the Grenadines | VCT |
| Ecuador | ECU | Samoa | WSM |
| Egypt | EGY | San Marino | SMR |
| El Salvador | SLV | Sao Tome and Principe | STP |
| Equatorial Guinea | GNQ | Saudi Arabia | SAU |
| Eritrea | ERI | Senegal | SEN |
| Estonia | EST | Serbia | SRB |
| Eswatini | SWZ | Seychelles | SYC |
| Ethiopia | ETH | Sierra Leone | SLE |
| Falkland Islands (Malvinas) | FLK | Singapore | SGP |
| Faroe Islands | FRO | Sint Maarten (Dutch part) | SXM |
| Fiji | FJI | Slovakia | SVK |
| Finland | FIN | Slovenia | SVN |
| France | FRA | Solomon Islands | SLB |
| French Guiana | GUF | Somalia | SOM |
| French Polynesia | PYF | South Africa | ZAF |
| French Southern Territories | ATF | South Georgia and the South Sandwich Islands | SGS |
| Gabon | GAB | South Sudan | SSD |
| Gambia | GMB | Spain | ESP |
| Georgia | GEO | Sri Lanka | LKA |
| Germany | DEU | State of Palestine | PSE |
| Ghana | GHA | Sudan | SDN |
| Gibraltar | GIB | Suriname | SUR |
| Greece | GRC | Svalbard and Jan Mayen Islands | SJM |
| Greenland | GRL | Sweden | SWE |
| Grenada | GRD | Switzerland | CHE |
| Guadeloupe | GLP | Syrian Arab Republic | SYR |
| Guam | GUM | Tajikistan | TJK |
| Guatemala | GTM | Thailand | THA |
| Guernsey | GGY | Timor-Leste | TLS |
| Guinea | GIN | Togo | TGO |
| Guinea-Bissau | GNB | Tokelau | TKL |
| Guyana | GUY | Tonga | TON |
| Haiti | HTI | Trinidad and Tobago | TTO |
| Heard Island and McDonald Islands | HMD | Tunisia | TUN |
| Holy See | VAT | Türkiye | TUR |
| Honduras | HND | Turkmenistan | TKM |
| Hungary | HUN | Turks and Caicos Islands | TCA |
| Iceland | ISL | Tuvalu | TUV |
| India | IND | Uganda | UGA |
| Indonesia | IDN | Ukraine | UKR |
| Iran (Islamic Republic of) | IRN | United Arab Emirates | ARE |
| Iraq | IRQ | United Kingdom of Great Britain and Northern Ireland | GBR |
| Ireland | IRL | United Republic of Tanzania | TZA |
| Isle of Man | IMN | United States Minor Outlying Islands | UMI |
| Israel | ISR | United States of America | USA |
| Italy | ITA | United States Virgin Islands | VIR |
| Jamaica | JAM | Uruguay | URY |
| Japan | JPN | Uzbekistan | UZB |
| Jersey | JEY | Vanuatu | VUT |
| Jordan | JOR | Venezuela (Bolivarian Republic of) | VEN |
| Kazakhstan | KAZ | Viet Nam | VNM |
| Kenya | KEN | Wallis and Futuna Islands | WLF |
| Kiribati | KIR | Western Sahara | ESH |
| Kuwait | KWT | Yemen | YEM |
| Kyrgyzstan | KGZ | Zambia | ZMB |
| Lao People's Democratic Republic | LAO | Zimbabwe | ZWE |
